# A systematic review of complementary feeding practices in South Asian infants and young children: the Bangladesh perspective

**DOI:** 10.1186/s40795-017-0176-9

**Published:** 2017-07-12

**Authors:** Logan Manikam, Alexandra Robinson, Jia Ying Kuah, Hrisheekesh J. Vaidya, Emma C. Alexander, George W. Miller, Kunjshri K. Singh, Victoria Dawe, Sonia Ahmed, Raghu Lingam, Monica Lakhanpaul

**Affiliations:** 1 Population, Policy & Practice, UCL Great Ormond Street Institute of Child Health, 30 Guilford Street, London, WC1N 1EH UK; 2 St George’s Hospital, Blackshaw Road, Tooting, London, SW17 0QT UK; 3 King’s College London GKT School of Medical Education, Guy’s Campus, London, SE1 1UL UK; 4 Imperial College School of Medicine, Faculty Building South Kensington Campus, London, SW7 2AZ UK; 5 Guy’s Hospital, Guy’s and St Thomas’ NHS Foundation Trust, Great Maze Pond, London, SE1 9RT UK; 6 Institute of Health & Society, Newcastle University, The Baddiley-Clark Building, Richardson Road, Newcastle upon Tyne, NE2 4AX UK

**Keywords:** Infant, Diet, Child, Nutrition, CF, Bangladesh

## Abstract

**Background:**

Sub-optimal nutrition among children remains a problem across South Asia (SA). Appropriate complementary feeding practices (CFP) can greatly reduce this risk. The primary objective of this systematic review (SR) of CF studies was to assess timing, dietary diversity, meal frequency and influencing factors in children under two in Bangladesh.

**Methods:**

Searches included English-language research published between January 2000 and June 2016 within MEDLINE, EMBASE, Global Health, Web of Science, OVID Maternity & Infant Care, BanglaJOL, Cochrane Library, CINAHL, POPLINE and WHO Global Health Library. Eligibility criteria: primary research concerning the adequacy of complementary feeding practices in South Asian children aged 0–2 years and/or their families. We excluded interventional papers and those focusing exclusively on breast-feeding. In total 45,712 titles and abstracts were screened against inclusion criteria, 860 of which received independent full text review by two reviewers. 36 papers relevant to Bangladesh were identified. The ‘EPPI-Centre Weight of Evidence Framework’ was used to objectively assess each study’s value in answering the review question. As per WHO Infant and Young Children Feeding Guidelines (IYCF), introduction of CF was assessed as the proportion of infants aged 6–8 months who received solid, semi-solid or soft foods. Search terms were: “children”, “feeding” and “Asians” with their derivatives. Two researchers undertook study selection, data extraction and quality appraisal.

**Results:**

Three cohort, 30 cross-sectional and 3 mixed methods studies were included. Despite adopting the WHO IYCF Guidelines, sub-optimal CF practices were found in many studies. Timely initiation of CF practices ranged from 24 to 83%. Achieved minimum dietary diversity ranged from 25% to 44% and minimum meal frequency from 33% to 81%. Influencing factors included maternal education, poor knowledge of CF practices and socioeconomic variables.

**Conclusions:**

This is the first systematic review to evaluate CF practices in Bangladesh. Despite adoption of the WHO IYCF guidelines, inadequate CFP remain in communities across Bangladesh.

**Trial registration:**

PROSPERO Registration No: CRD42014014025.

**Electronic supplementary material:**

The online version of this article (doi:10.1186/s40795-017-0176-9) contains supplementary material, which is available to authorized users.

## Background

Undernutrition including stunting and suboptimal breast feeding accounts for 45% of all childhood deaths [1]. It is estimated that 70% of the world’s stunted children live in Asia with Bangladesh having the second highest rate of child undernutrition in the world [1, 2].

The WHO defines complementary feeding as: “The process starting when breast milk alone is no longer sufficient to meet the nutritional requirements of infants, and therefore other foods and liquids are needed, along with breast milk” [3].

Complementary feeding therefore focuses on bridging the gradual transition between 6 and 24 months from exclusive breastfeeding to solid foods eaten by the whole family alongside breastfeeding. Poor CFP have been linked to increased risks of respiratory and gastrointestinal infections alongside mortality [4, 5]. Only 71% of Bangladeshi infants consume appropriate complementary foods by 6 to 8 months of age and often ‘[fall] off the growth curve’ once CF begins [2, 6]. Therefore, it is vital that WHO recommendations on CF are widely adopted [7]. Appropriate CF requires sufficient household food availability and adequate nutritional knowledge application by caregivers [8]. While vast numbers of studies exist on BF practices, CF remains less extensively studied.

The 2010 WHO Infant and Young Children Feeding (IYCF) guidelines, an internationally ratified framework adopted in Bangladesh, emphasises that as a global public health recommendation, infants should be exclusively breastfed for the first 6 months of life to achieve optimal growth, development and health [9]. Thereafter, infants should receive safe and nutritionally adequate complementary foods while breastfeeding continues for up to 2 years of age or beyond.

With no previously published systematic review identified, we aimed to assess the adequacy of CFPs based on IYCF recommended minimum dietary diversity and meal frequency, timing of introducing CF and barriers and promoters influencing CFPs amongst South Asian (SA) children.

To limit the scope of our review, we focused on SA families residing in Bangladesh, India, Pakistan and Developed Countries (DC). This would inform future work in developing and assessing the effectiveness of culturally appropriate interventions to improve CFPs across these communities.

## Methods

Due to the vast number of publications identified, this review summarises publications of CFP in SA families in Bangladesh only with concurrent reviews summarising publications of CFP in SA families in DC, Pakistan and India respectively. This was done in order to allow the reviews to go into sufficient depth on the details of CFP in each country. PRISMA guidelines/methodology were adhered to in this review.

### Eligibility criteria

Studies were included if they met the following criteria:

- Participants: Children aged 0–2 years, parents, carers and/or their families
- Outcomes: Adequacy of complementary feeding (based on minimum dietary diversity and meal frequency), timing of introduction of CF and barriers/promoters to incorporating WHO recommended CFP
- Language: Studies published in English, or with translation available
- Year: Published from 2000 or later

In the IYCF indicators, introduction of CF is assessed as the proportion of infants aged 6–8 months who receive solid, semi-solid or soft foods. In contrast, minimum dietary diversity is assessed by the proportion of 6–23 months of age who receive foods from 4 or more food groups. The 7 WHO IYCF recommended food groups consist of [7];

1. Grains, roots and tubers (G)
2. Legumes and nuts (L)
3. Dairy products (e.g. milk, yoghurt, cheese) (D)
4. Flesh foods (e.g. meat, fish, poultry, and liver/organ meats) (F)
5. Eggs (E)
6. Vitamin A rich fruits and vegetables (A)
7. Other fruits and vegetables. (V)

Whilst the consumption of iron rich or iron fortified foods is commonly assessed as a separate IYCF indicator, this was incorporated within dietary diversity for ease of interpretation.

Finally, minimum meal frequency (MMF) was assessed by the proportion of breastfed and non-breastfed children 6–23 months of age who receive solid, semi-solid, or soft foods (but also including milk feeds for non-breastfed children) according to the minimum number of times or more per day; 2 for 6–8 months, 3 for 9–23 months and 4 for 6–23 months (if not BF) [8].

Due to the nature of the topic, all study types (qualitative, quantitative or mixed) were included to ensure the diversity of evidence was captured and summarised to be of relevance to both policy makers and health and social care professionals. We excluded studies focusing on exclusive breastfeeding and interventional studies, as such studies did not reflect the focus of this review, mainly CF practices.

### Information sources

A search strategy was devised to search the following databases: MEDLINE, BanglaJOL, EMBASE, Global Health, Web of Science, OVID Maternity & Infant Care, The Cochrane Library, POPLINE, CINAHL and WHO Global Health Library. Searches were conducted in December 2014 and updated in June 2016.

Members of electronic networks on @jiscmail.ac.uk including minority-ethnic-health and networks (eg. South Asian Health Foundation) developed from the Specialist Electronic Library for Ethnicity and Health were contacted to request any additional or unpublished material from members of the networks. Bibliographies of included articles were also hand-searched for possible additional publications.

### Search strategy

The search strategy included the terms “feeding”, “South Asian” (including terms specifying all major subgroups) and “children”. For example, the search strings used for MEDLINE were:

- Term 1: Children <2 years
  Infant OR Baby OR Babies OR Toddler OR Newborn OR Neonat* OR Child OR Preschool OR Nursery school OR Kid OR Pediatri* OR Minors OR Boy OR Girl
- Term 2: Feeding
  Nutritional Physiological Phenomena OR Food OR Feeding behavior OR Feed OR Nutrition OR Wean OR fortif* OR Milk
- Term 3: Asians
  Ethni* OR India* OR Pakista* OR Banglades* OR Sri Lanka OR Islam OR Hinduism OR Muslim OR Indian subcontinent OR South Asia

### Study selection and data extraction

**Fig. 1:**
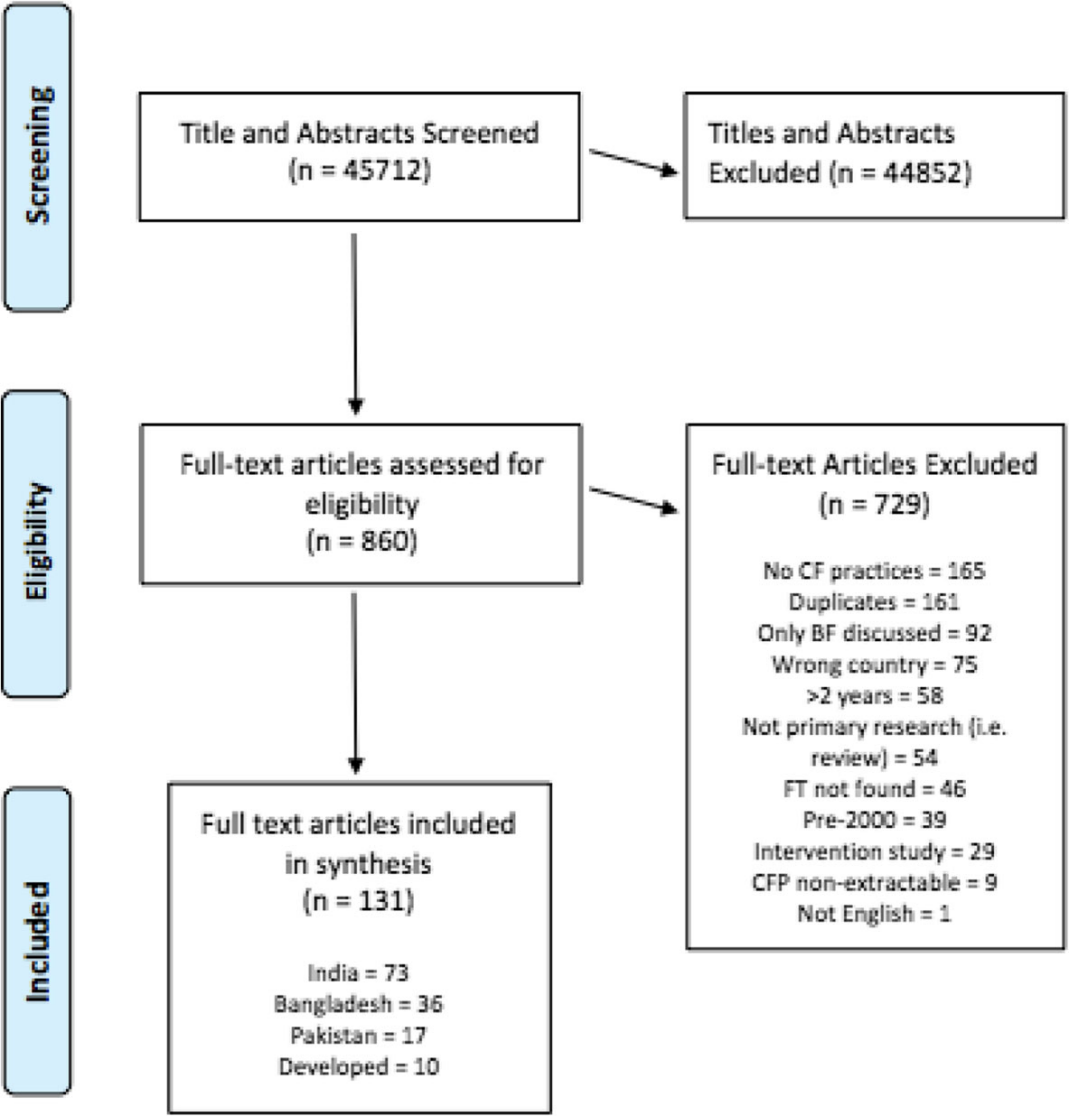
PRISMA Flow Diagram – Study Screening Process

In total, 45,712 titles and abstracts were screened against inclusion criteria. Two reviewers assessed these papers independently with conflicts resolved by discussion with the team. In view of the large number of articles deemed eligible for full-text review, articles published before the year 2000 were excluded, ensuring that included papers were strongly focused on current practice, closely preceding and also following the 2010 WHO guidelines. In total, 44,852 titles and abstracts were excluded, as shown in Fig. 1.

This left 860 potentially eligible full text articles describing CF practices in SA children, which were independently reviewed by two reviewers. 136 full text articles were ultimately extracted of which 36 papers were relevant to Bangladesh.

Data was extracted by a single reviewer using a piloted modified worksheet including: country of study; study type; study year; study objectives; population studied, eligibility criteria and illness diagnosis; study design; sampling; data collection and analysis; feeding behaviours; adequacy of CF practices; timing of initiation of CF; comments to aid interventional development; weight of evidence. A second member of the research team checked each extraction. Extractions were then double-checked to ensure accuracy.

### Result synthesis

The eligible studies tended to address very broad research questions, were conducted using qualitative and/or quantitative and/or descriptive methods, reported on diverse outcome measures and were not presented following standardised reporting guidelines (e.g. STROBE for observational studies or COREQ for qualitative research). Meta-analyses were therefore not undertaken.

To standardise study classifications, the formal definitions below were utilised and applied:

1. Cohort study; An observational study in which a group of patients are followed over time. These may be prospective or retrospective.
2. Cross sectional study; An observational study that examines the relationship between health-related characteristics and other variables of interest in a defined population at one particular time.
3. Mixed methods; A study that combines both quantitative and qualitative methodology.

In view of the considerable heterogeneity in studies identified in terms of methods, participants, interventions and outcomes, a narrative approach to synthesis was utilised using guidance developed from the University of York Centre for Reviews and Dissemination (CRD) and the Economic and Social Research Council (ESRC) [10, 11].

The evidence reviewed is presented as a narrative report with results broadly categorised following IYFP indicators on; (1) adequacy of CFP comprising of dietary diversity, meal frequency, timing of introduction of CFP and consumption of iron-rich foods and (2) barriers/promoters influencing CF practices.

Barriers were defined as obstacles or impediments to achieving correct CFP whilst promoters were defined as supporters to achieving correct CFP. These were subcategorized into factors influencing at the family (e.g. family members), and organizational level (e.g. health care providers, hospitals, political bodies).

### Quality assurance

The Centre for Reviews and Dissemination (CRD) guidance emphasises the importance of using a structured approach to quality assessment when assessing descriptive or qualitative studies for inclusion in reviews. However, it acknowledges the lack of consensus on the definition of poor quality with some arguing that using rigid quality criteria lead to the unnecessary exclusion of papers [10].

In our review, the EPPI-Centre Weight of Evidence Framework was used for objective judgements about each study’s value in answering the review question. It examines three study aspects: Quality of Methodology, Relevance of Methodology and Relevance of Evidence to the Review Question and categorises them to Low, Medium or High. An average of these weightings is taken to establish the study’s Overall Weight of Evidence. Studies with an Overall Weight of Evidence of Low are still included in the table of included studies but not discussed further within the results and discussion. This was performed by two independent reviewers, with additional arbitration by other team members where required [12].

## Results

### Study and participant characteristics

**Fig. 2:**
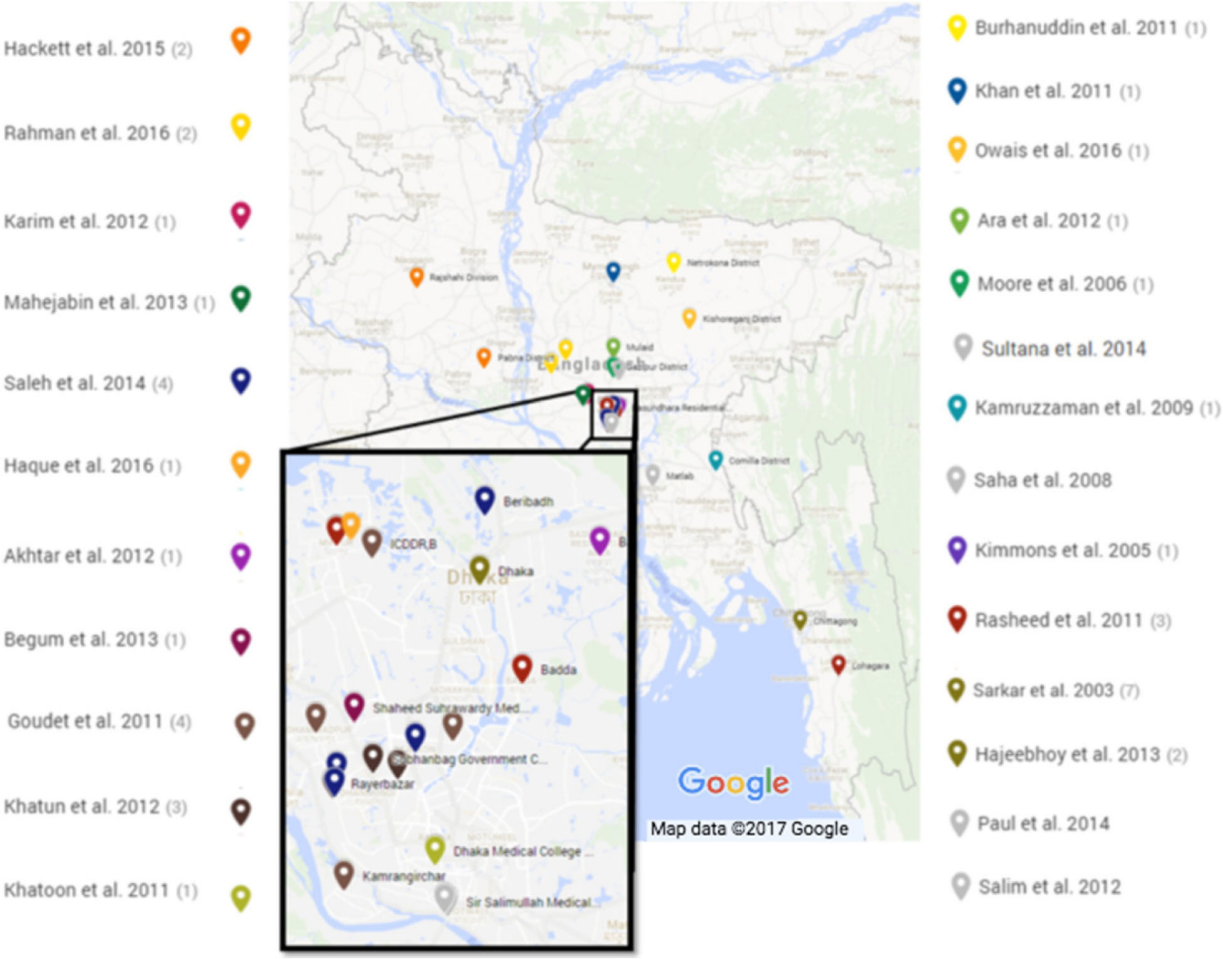
Map of included study locations (where available). Map courtesy of Google Maps. Map data ©2017 Google

The 36 studies consisted of 30 cross-sectional, 3 cohort and 3 mixed methods studies. Additional file 1: Table S1 denotes a summary of all the included studies. Figure 2 illustrates the study locations of 25 of the 36 included studies which provided mappable locations.

Table 1 presents the Weight of Evidence awarded to each of the studies. The core narrative themes extracted from the papers are presented under the headings; adequacy and factors influencing CFP. The former is categorised further into dietary diversity, meal frequency, timing, iron fortification and food hygiene.

**Table 1:** Weight of Evidence Awarded to Each Study

Studies	Weight of Evidence A	Weight of Evidence B	Weight of Evidence C	Weight of Evidence D
	Quality of Methodology: The accuracy, coherency and transparency of evidence.	Relevance of Methodology: The appropriateness of the methodology for answering the review question.	Relevance of evidence to the review question: The relevance of the focus of the evidence for answering the review question.	Overall weight of evidence: Overall assessment of the extent to which the study provides evidence to answer the review question
Akhtar et al. 2012 [22]	Low	Medium	Medium	Medium
Ara et al. 2012 [23]	Low	High	Medium	Medium
Begum et al. 2013 [21]	Low	Medium	Medium	Medium
BurhanUddin et al. 2011 [20]	Medium	Medium	High	Medium
Das et al. 2008 [45]	Medium	Medium	Low	Medium
Faruque et al. 2008 [2]	Medium	Low	Medium	Medium
Goudet et al. 2011 [25]	Medium	Medium	Low	Medium
Goudet et al. 2011 [24]	Medium	Medium	Medium	Medium
Hackett et al. 2015 [26]	High	Medium	Medium	Medium
Hajeebhoy et al. 2013 [44]	Low	Medium	Low	Low
Hanif et al. 2013 [19]	High	Medium	High	High
Haque et al. 2016 [27]	Medium	Medium	Medium	Medium
Helen Keller International 2009 [18]	High	Medium	Medium	Medium
Kabir et al. 2012 [6]	High	High	High	High
Kamruzzaman et al. 2009 [28]	Low	Medium	Medium	Medium
Karim et al. 2012 [29]	Low	Low	Medium	Low
Khan et al. 2011 [30]	Medium	Medium	Medium	Medium
Khatoon et al. 2011 [17]	Medium	Medium	High	Medium
Khatun et al. 2012 [46]	Medium	Medium	Medium	Medium
Kimmons et al. 2005 [31]	High	Medium	Medium	Medium
Mahejabin et al. 2013 [32]	Medium	Medium	Medium	Medium
Mihrshahi et al. 2010 [41]	Medium	High	High	High
Moore et al. 2006 [33]	Medium	Medium	Medium	Medium
Islam et al. 2008 [34]	Medium	Medium	Low	Medium
Nguyen et al. 2013 [16]	High	High	High	High
Owais et al. 2016 [15]	High	High	High	High
Paul et al. 2014 [35]	Medium	Medium	Medium	Medium
Rahman et al. 2016 [36]	High	High	High	High
Rasheed et al. 2011 [14]	Medium	High	High	High
Rawat et al. 2014 [13]	High	Medium	Medium	Medium
Saha et al. 2008 [37]	Medium	High	High	High
Saleh et al. 2014 [40]	Medium	Medium	Medium	Medium
Salim et al. 2012 [38]	Low	Medium	Medium	Medium
Sarkar et al. 2003 [42]	Low	Medium	Medium	Medium
Sultana et al. 2014 [39]	Medium	Medium	High	Medium
Zongrone et al. 2012 [8]	Medium	Medium	Medium	Medium

### Adequacy of complementary feeding

As per the WHO IYCF indicators, adequacy of CFP is assessed according to minimum dietary diversity (MDD), meal frequency and timing of introducing CFP. These are detailed in the subheadings below.

#### Minimum dietary diversity

To achieve MDD, a child aged 6–23 months must receive food from at least four of the seven WHO-defined food groups every day. Dietary diversity was explored in 11 studies [6, 8, 13–21], with 4 using the seven WHO IYCF food groups to quantify MDD [14–16, 19]. Their data suggests a declining trend in MDD achievement between 2007 and 2011, with current attainment around 25%. Diversity of maternal diet was positively associated with child MDD, and mothers tend to consume a larger range of food groups than their children [15, 16].

Three studies use data from the nationwide Bangladesh Demographic Health Surveys (BDHS), which showed that in 2007 MDD was achieved in approximately 44% of children [6, 8]. The 2007 BDHS combined groups M and E into a single “meat, fish, and eggs” group; this figure may therefore underestimate the true value. In the 2011 BDHS the reported rate had fallen to 25% [19]. A large cohort study conducted in rural sub-districts in 2011 provides an even lower number; Owais et al. (Weight of Evidence = High (WOE = H)) interviewed 2073 mother-child pairs over 9 months, and found that just 16% of children received the necessary four food groups per day to satisfy MDD [15]. These figures are partially supported by two further studies on rural subdistricts in 2009 and 2010; Rasheed et al. (WOE = H) collected information on 98 households not involved in any nutrition program in 2009 and found that 24% achieved MDD [14], whereas the Alive and Thrive baseline survey by Nguyen et al. (WOE = H), conducted in 2010 on 1211 infants, found that 31.1% achieved MDD [16].

The food used for complementary feeding could be categorised into WHO IYCF food groups for 27 of the 36 studies. In general, foods were heterogeneously classified; many authors combined or added to the WHO food groups, and some did not specify the ingredients of categories - eg. “thick” foods, “gruel”, “family foods”, or “hotchpotch”.

All 27 studies reported use of “grains, roots, and tubers” (group G) other than Khatoon et al. (Weight of Evidence = Medium) (WOE = M)), which used different food groupings including “carbohydrate-rich foods” [6, 8, 13–18, 20, 22–39]. Similarly, 24 of 27 papers referred to the use of “legumes and nuts” (group L), mostly in the form of lentils [6, 8, 13–16, 18, 20, 22, 24–33, 35–39]. These two groups were frequently encountered in khichri, a preparation of rice with lentils. This popular weaning food provides two WHO IYCF food groups (G & L), and is at times combined with vegetables (V) [14, 25, 33, 36], meat (F) [14, 33] or eggs (E) [14, 33].

In Bangladesh, eggs (group E) were the least commonly used group for complementary feeding, with only 16 studies reporting their use in their populations [6, 8, 13–16, 18, 22, 27, 28, 31, 33, 35, 36, 38, 39]. Even among those that did, use was frequently low; Owais et al. reported that neither the mother nor the child ate eggs in 79% of the 2073 pairs they interviewed [15]. Similarly, dairy products were reported to be used by 23 studies [6, 8, 15–17, 20, 22–32, 35–39], but by less than 40% of mothers in studies by Owais et al. [15], Khatoon et al. [17], and Burhanuddin et al. [20].

Complementary feeding with fruit and vegetables (groups V and A) was identified in 23 studies [6, 8, 13–18, 20, 22–31, 33, 35, 36, 38, 39], and reported use varied widely - Sultana et al. (WOE = M) reported that only 1.4% of infants consumed leafy green vegetables daily, whereas Owais et al. reported that 43% of infants consumed non-Vitamin A rich fruit and vegetables [15, 39]. Flesh foods (e.g. meat, fish, poultry, and liver/organ meats) were identified in 21 studies [6, 8, 13–18, 22, 23, 26–31, 33, 35, 36, 38, 39], with 16 particularly identifying fish as being used for CF, and 12 identifying “meat”.

While maternal and child dietary diversity was associated, the proportions of each food group differed. Both Owais et al. and Nguyen et al. noted that mothers were less likely to provide flesh foods (F) and fruit and vegetables (A, V) to their children, even if these featured in the maternal diet [15, 16]. Hackett et al. (WOE = M) described that while vegetables were perceived to be good complementary foods, just 14% of the women interviewed suggested feeding meat, fish, or other animal-source foods to young children [26].

#### Iron rich foods

As explored in the previous section, many studies mentioned meat being used for CF, but type of meat was not often specified. In Rawat et al. (WOE = M) 56% of infants were noted to be iron deficient in 20 rural sub districts. Fish was the most commonly consumed iron-rich food (i.e. by 19% of infants), whilst less than 14% of infants consumed plants as a source of iron [13]. Zongrone et al. (WOE-M) found that 51.46% of infants consumed iron-rich foods generally [8], and Rasheed et al. (WOE = H) found that 10% of infants 6–23 months consumed recommended levels of iron [14].

#### Food preparation

Six of 36 studies discussed food preparation. Two studies noted that infants’ foods were rarely prepared separately from the rest of the family’s foods [14, 36], but Haque et al. (WOE = M) said most mothers prepared infants’ foods separately [27]. Goudet et al. (WOE = M) observed that when women’s homes were not supplied with gas, special food preparation for infants was not possible [25]. Hackett et al. (WOE = M) found that 67% of participants thought safe preparation of infants’ foods was important, covering appropriate washing, cooking and covering [26]. Finally, Paul et al. (WOE = M) found that blenders were used for preparing CF by 7.55% of sampled mothers [35].

#### Food hygiene

Food hygiene was explored in 5 of 36 studies. In Goudet et al. (WOE = M), lack of appropriate steps to ensure adequate hygiene before and during feeding was noted in Dhaka slums [24]. Handwashing was discussed in three studies - Saleh et al. (WOE = M) noted that 26% of mothers didn’t properly clean their hands and utensils before feeding with 33% of children not washing their hands [40]. Hackett et al. (WOE = M) found that although around a third of 70 women communicated the importance of hygiene in preparing and storing foods, only four mentioned washing hands before feeding [26]. Sultana et al. (WOE = M) found that although 76% of 71 mothers said they washed hands properly before feeding, only 11.3% washed both hands and utensils properly [39]. Interviews with 12 mothers conducted by Rahman et al. showed all mothers were knowledgeable about covering food to avoid contamination, and 10/12 mentioned washing plates and utensils [36].

#### Meal frequency

Meal frequency was assessed in 14 of 36 studies. Minimum meal frequency (MMF) rates varied by age and breastfeeding status. Calculated mean feeds ranged from 2.4 to 3.2. Information on frequency for each study is included in Additional file 1: Table S1.

In Kabir et al. (WOE = H), overall 81.06% of 6–23 month-olds met MMF requirements. MMF rates increased with age; 66.17% of 6–11 months olds met MMF, rising to 77.78% at 12–17 month olds, and 93.55% at 18–23 month olds [6]. Owais et al. (WOE = H) also found generally high meal frequencies, with 74% of infants receiving over 4 meals in the previous 24 h [15]. Rasheed et al. (WOE = H) found that 59.0% of 6–11 month olds, and 59.8% of 12–24 month olds, were fed at the recommended frequency for breastfed children [14]. This was similar to Paul (WOE = M) who found that frequency was appropriate in 53.5% [35]. In Saleh et al. (WOE = M), frequency rates varied by age and exclusive breastfeeding status; they found that 33% of non-exclusively breastfed 6–8 month olds were fed 3 times per day, compared to 81% of exclusively breastfed 12–23 month olds [40].

Three studies calculated mean daily feeds - Kimmons et al. (WOE = M) found that the mean number of daily feeds was 2.6 - this compared with 2.4 in Khatoon et al. (WOE = M) and 3.2 in Munirul Islam (WOE = M) [17, 31, 34]. Sultana et al. (WOE = M) examined frequency of feeding of various foods; they found that Dal was the most fed daily food, being consumed >1 per day by 19.7% of included children [39].

#### Timing of introducing CF

The timing of the introduction of CF was investigated in 26 of 36 studies. CF initiation was most commonly reported between 6 and 9 months (21 studies [2, 6, 8, 14, 15, 18–23, 26–28, 30, 32, 37–41]) followed by 3–6 months (19 studies [14, 15, 18, 20–24, 26–30, 33, 37, 39–42]). Four [14, 18, 22, 37], three [27, 28, 37] and two [28, 33] studies each noted that CF was started younger than 3 months, at between 9 and 12 months, and after 12 months respectively.

Timely initiation at 6 months was often reported, but proportions ranged widely. Moore et al. (WOE = M) reported that the mean age for initiation was 6.8 months [33]. Zongrone (WOE = M) and Sultana (WOE = M) reported that CF was timely in around 80%, and 83.1%, respectively [8, 39]. This contrasted with Paul (WOE = M), who reported that timely initiation at 6 months occurred in 48.4% [35]. For Khan et al. (WOE = M) timing was appropriate for 35.8%, and for Salim et al. (WOE = M) this figure was 24% [30, 38].

Early initiation rates were sometimes low and sometimes moderate, ranging from 13.2% by 5 months in Saha et al. (WOE = H), 16.9% in Sultana et al., and 19% at 4–5 months in Akhtar et al. (WOE = M) to higher rates of 44.5% and 47.8% reported respectively by Khan et al. and Salim et al. [22, 30, 37–39]. Regarding subgroups, Sarkar et al. (WOE = M) found that 58% of foster children were fed CF before 4 months, compared to 14% in the non-fostered comparison group [42]. Burhan Uddin et al. (WOE = M) did not find a difference in appropriate initiation when comparing tribal and non-tribal mothers, with 87% of Garo and 84% Non Garo mothers starting CF before 6 months of age, with 13% and 16% respectively starting CF after 6 months of age [20].

Regarding the extremes of very early or late CF, Kamruzzaman et al. (WOE = M) noted that CF was not initiated until after 12 months by 1 of 54 infants in their sample, and Moore et al. also found initiation took place between 12 and 14 months for a small number of infants [28, 33]. Regarding very early CF, Akhtar et al. described 2.4% of their sample commencing CF at 2–3 months, and Saha et al. found that 0.4% of their sample were given CF before 2 months of age [22, 37]. Rasheed et al. (WOE = H) described women who fed CF at 2–3 months “when the baby cried due to hunger” and Helen Keller International (WOE = M) described gruel being given before 3 months [14, 18].

#### Sources of advice for feeding

Six of 36 studies described advice providers for CF. The most commonly mentioned source of feeding advice was healthcare professionals, including doctors and hospital staff (5 studies [14, 17, 20, 35, 43]). The next most common source was from family members (4 studies [14, 20, 26, 35]). The media, including newspapers, radio and television were mentioned by 3 studies [20, 35, 44] as were neighbours [14, 20, 35]. Other sources included traditional birth attendants (1 study [14]), religion (1 study [26]), and the nutritionist (1 study [20]). Paul et al. (WOE = M) surveyed proportions who received advice from various sources, and found the most popular source was relatives (for 25%), followed by qualified doctors (15.3%), neighbors (14.5%), and the mother-in-law (13.5%) [35]. The husband, television, other family members, other health workers, friends and newspapers were also mentioned [35].

### Factors associated with IYCF practices

We identified numerous factors that influenced CFP. These are summarised in Table 2 as either a Barrier or Promoter and subcategorized as either acting at family or organizational level. Due to conflicting study findings, factors may appear as both a Barrier and Promoter. 16 promoters and 22 barriers influencing CFP were identified.

**Table 2:** Factors influencing CF

Family level			
Promoters	Study number	Barriers	Study number
Father’s occupation	1 study [6]	Father’s occupation	1 study [6]
Mother with secondary or primary education	7 studies [6, 16, 23, 31, 38, 39, 45]	Mother with poor education	9 studies [6, 21, 23, 35, 36, 38, 39, 45, 41]
Knowledge on appropriate CFP recommendations and benefits	3 studies [14, 32, 36]	Father with poor education	1 study [6]
The practice of responsive feeding; applying the principles of psychosocial care during feeding e.g. strategies to overcome poor child appetite.	1 study [33]	Lack of knowledge of CF	8 studies [14, 24, 26, 32, 35, 40, 42, 46]
Higher number of food groups consumed in maternal diet	2 studies [15, 16]	Maternal death leading to fostering of child	1 study [42]
Education on health nutrition by health workers	1 study [35]	Psychosocial care during feeding, Mothers’ strategies to overcome poor child appetite(e.g. force feed child refusing CF).	3 studies [26, 33, 40]
Mothers produced sufficient milk to feed baby for over 6 months	1 study [35]	Limited engagement of mother with e.g. TV, radio, newspapers.	1 study [6]
Mother engages with media sources; newspapers, radio, TV	1 study [6]	Cultural factors and taboos	3 studies [14, 26, 35]
Child in question is male	2 studies [26, 37]	Family members influence CFP (Lack of support for appropriate CFP, advice, decision making, family dimensions etc.)	3 studies [14, 26, 35]
Child’s taste and behavioural response to appropriate CF given (e.g. perceived preference).	1 study [14]	Higher maternal parity	3 studies [16, 36, 45]
		Maternal employment	2 studies [24, 36]
		Perceived /actual inadequacy of Mother’s breast milk supply to breastfeed for 6 months.	5 studies [14, 24–26, 35]
		Time allocation of mother to household chores /work reduces time to address CFP	4 studies [14, 24, 32, 36]
		Child’s taste and behavioural response to CF given (e.g. refuses/doesn’t cry when certain foods offered)	2 studies [14, 35]
		Illness of parent	2 studies [24, 32]
Organisational level			
Promoters	Study number	Barriers	Study number
Residence in urban area	2 studies [6, 41]	Residence in rural area	3 studies [6, 45, 41]
Interventions (e.g. health and Nutrition educators) advocating and stimulating families to practice WHO recommendations on CFP	1 study [2]	Bureaucratic policies on IYCF practices and outreach of information on appropriate CFP to public.	1 study [44]
Mother having higher number of antenatal check ups	3 studies [6, 45, 41]	Mothers having no antenatal check-ups	1 study [6]
Higher household food security	2 studies [15, 37]	Living in areas that flood	2 studies [24, 25]
Household wealth index rich or richest	4 studies [6, 37, 45, 46]	Household wealth index poor or poorest.	7 studies [6, 22, 24, 25, 42, 45, 46]
Use of local officials to promote food already in households to be used as CF	1 study [14]	Lower household food security	4 studies [15, 24, 37, 44]
		Lack of provision of maternity leave	1 study [44]

#### Promoters

Eight studies identified promoters at the organizational level. Promoters were: Residence in urban area, Interventions (e.g. health and nutrition educators) advocating and stimulating families to practice WHO recommendations on CFP, Mother having higher number of antenatal check-ups, Higher household food security, Higher household wealth index, Presence of local officials to promote food already in household to be used as CF.

The most common promoter of CF at the organisational level was higher household wealth index, identified in 4 studies by Kabir et al. (WOE = H), Saha et al. (WOE = H), Das et al. (WOE = M), and Khatun et al. (WOE = M) [6, 37, 45, 46].

Fifteen studies identified promoters at a family level. Promoters were: Father’s occupation, Mother with secondary or primary education, Knowledge on appropriate CFP recommendations, The practice of responsive feeding (e.g. strategies to overcome poor child appetite), Higher number of food groups consumed in maternal diet, Education on health nutrition by health workers, Mothers produced sufficient milk to feed baby for over 6 months, Mother engages with media sources, Child in question is male, Child’s taste and behavioural response to appropriate CF given (e.g. perceived preference).

The most common promoter of good CF at the family level was levels of maternal education, identified in seven studies by Kabir et al., Nguyen et al. (WOE = H), Kimmons et al. (WOE = M), Ara et al. (WOE = M), Das et al., Salim et al. (WOE = M), and Sultana et al. (WOE = M), [6, 16, 23, 31, 38, 39, 45]. It is notable that two studies found that children were likely to receive improved CF if they were male [26, 37].

#### Barriers

Eleven studies identified barriers at the organizational level. Barriers were: Residence in rural area**s,** Bureaucratic policies on IYCF practices and outreach of information on appropriate CFP to public, Mothers having no antenatal check-ups**,** Living in areas that flood, Low Household wealth index, Lower household food security, Lack of provision of maternity leave.

The most commonly identified barrier at the organisational level was a poor household wealth index, by seven studies - Kabir et al. (WOE = H), Akhtar et al. (WOE = M), Das et al. (WOE = M), Goudet et al. (WOE = M), Goudet et al. (WOE = M), Khatun et al. (WOE = M), Sarkar et al. (WOE = M) [6, 22, 24, 25, 42, 45, 46]. Two studies identified that flooding poses a barrier to good CF practices [24, 25].

Eighteen studies identified barriers at a family level. Barriers were: Father’s occupation, Mother with poor education, Father with poor education, Lack of knowledge on CFP, Maternal death leading to fostering of child, Psychosocial care during feeding, Mothers’ strategies to overcome poor child appetite(e.g. force feed child refusing CF), Limited engagement of mother with media, Cultural factors and taboos, Lack of support by family members influence CFP, Higher maternal parity, Perceived /actual inadequacy of Mother’s breast milk supply to breastfeed for 6 months, Time allocation of mother to household chores, Child’s taste and behavioural response to CF given, and Illness of parent.

The most commonly identified barriers at the family level were mother with poor education (9 studies [6, 21, 23, 35, 36, 38, 39, 45, 41]) and lack of knowledge of CF (8 studies [14, 24, 26, 32, 35, 40, 42, 46]). It is notable that two studies found that maternal employment was a barrier to good CF [24, 36].

## Discussion

To our knowledge, this is the first systematic review to assess CFP in Bangladesh. We identified that in many SA families in Bangladesh, WHO IYCF standards on minimum dietary diversity, meal frequency, and timing of introducing CF were not being met.

### Implications of key findings

Dietary diversity was found to be inadequate for many children with some noting poor consumption of dairy, eggs, fruit and vegetables [15–17]. Whilst raising awareness on MDD by improving knowledge is likely to be beneficial, the delivery mode of such an intervention needs to be established [6]. With evidence of an increasing prevalence of iron deficiency anaemia within 3 months of CF initiation, supporting mothers’ use of iron rich or fortified foods is essential with some advocating the use of universal distribution of iron fortified foods for low households [13, 18, 19, 47].

Levels of maternal education have been identified as an influence on CFP. Some have argued that as most rural girls have become wives prior to the age of 18 despite laws preventing this, impeding schooling, tougher legal enforcement is necessary [41]. In contrast, some have argued that social and financial empowerment of future mothers is a more effective upstream intervention alongside better education and caregiving support [13]. This may be particularly important for working women, given that two studies in this review identified maternal employment as a barrier to appropriate CF [24, 36].

Household wealth indexes were identified by several studies as associated with CF practices. Long term interventions such as improving secondary education and financial aid may be beneficial in improving household wealth indices [24].

Improved education on appropriate CFP and household wealth is unlikely to lead to practice changes in isolation; behavioural change interventions addressing local cultural beliefs may be equally necessary to improve CFP [45]. As this study identified that family members can influence CF, interventions should be aimed at wider communities and relatives, not just parents [14, 26, 35]. Alongside this, improved basic amenities in flood prone, rural and slum areas is necessary to reduce the morbidity associated with gastrointestinal infections secondary to poor food hygiene [24, 37].

#### Strengths and limitations

The strengths of our systematic review are derived by searching 10 databases utilising broad search strings, performing an updated search in June 2016 and having two reviewers undertake study selection, data extraction and quality assessment. By identifying factors that promote and are barriers to CFP, this study could assist with the development of interventions to improve CFP in future.

A key limitation of this paper is the exclusion of a large number of papers published before the year 2000 at full text review. Although this limited the range of the search, it allowed the final paper to be focused on current practice especially in the context of WHO IYCF recommendations. Other limitations include excluding papers which solely focused on children over 2 years, where CFP described in their younger years may have been missed, and papers not published in English, which would have both added to the diversity of CFP described.

A further limitation is that in several studies where there was overlap between children under and over 2 years and/or SAs by Indian, Pakistani and Bangladeshi origin, CFPs described and attributed to the whole study population maybe incorrect. Furthermore, in the Diversity section, we did not develop a composite measure of the quantities of the foods used, only the frequency with which they appeared in the studies.

Whilst we excluded interventional studies which may have described CFP in their study population, this is unlikely to be the primary focus of such studies and therefore unlikely to significantly affect our systematic review.

Finally, whilst we attempted to contact numerous authors to identify relevant grey literature for our review, due to the breadth and depth of the field of nutritional research this study is unlikely to be exhaustive with publication bias likely to be present.

## Conclusion

This systematic review has highlighted CFPs covering dietary diversity, frequency, timing, hygiene, preparation, and the factors that influence them, which may provide guidance for development of context-tailored interventions. Despite adoption of the WHO IYCF guidelines, inadequate CFP remain in SA communities across Bangladesh. Increased awareness of this issue can pave the way for improvements in the future.

## Additional file

Additional file 1: Table S1. Description of data: A summary of all included studies. (DOCX 57 kb)

## Abbreviations

BF: Breast feeding
CF: Complementary feeding
CFP: Complementary feeding practices
CRD: Centre for Reviews and Dissemination
DC: Developed countries
ESRC: Economic and Social Research Council
IYCF: Infant and Young Children feeding
MDD: Minimum dietary diversity
MMF: Minimum meal frequency
SA: South Asia
SR: Systematic review
WHO: World Health Organisation
WOE = H: Weight of evidence = High
WOE = M: Weight of evidence = Medium
