# Supplementary material for: A systematic review of complementary feeding practices in South Asian infants and young children: the Bangladesh perspective
Source: BMC Nutr. 2017 Jul 12;3:56. doi: 10.1186/s40795-017-0176-9 (PMC7050712; doi:10.1186/s40795-017-0176-9)
Supplement: Supplementary file 1 — Description of data: A summary of all included studies. (DOCX 57 kb) [file 40795_2017_176_MOESM1_ESM.docx]

**Additional file – Table S1**

| **Table S1 – A summary of all included tables** | | | | | |
| --- | --- | --- | --- | --- | --- |
| **Author** | **Study type** | **Location** | **Population** | **Sample size** | **Adequacy of and factors influencing CFP** |
| Akhtar et al. 2012 [22] | Cross-sectional | One slum near Basundhara residential area under Dhaka City Corporation, Bangladesh. | Apparently healthy child aged between 6 to 23 months | 125 children | Diversity: Foods used for CF includedbarley, biscuit, cerelac, family food (rice, lentil, vegetables; only 5 fed with fish/meat/egg), kichuri, noodles, powder milk, rice with milk.  Timing: 64% of mothers started CF at 6-7 months, while 19% started at 4-5 months, 2.4% at 2-3 months, and 14% started >7 months.  Factors: Poverty limited foods available for CF. |
| Ara et al. 2012 [23] | Cross-sectional | Rural community of Mulaid, Gazipur, Bangladesh. | Mothers, having children between 6 months and 2 years, selected through cluster sampling | 227 mothers of children aged 6-24 months. | Diversity: Rice, fruit & veg, fish, hotchpotch, milk and milk protein were used for CF.  Timing: In 42.3% CFP started at an appropriate age. Early in 33.5%. Late in 24.2%.  Factors: Barriers - Poor education/illiteracy appropriate CFP 45% in educated mothers, 33% in illiterate mothers. Promoters - Education/Literacy |
| Begum et al. 2013 [21] | Cross-sectional | Paediatrics outpatients department of Shahid Surhawardy Medical College and Hospital, Dhaka, Bangladesh. | Babies attended in OPD of more than six months and less than one year age group of both sex accompanied by their mother were enrolled in this study | 250 infants | Diversity: Study describes consistency of food - thick or thin, and then CF "amount" and "composition" as appropriate or not  Timing: CF was too early before six months in 29.6%(74/250) cases  Factors: education status of the mother was mostly less than 10 class which might impact on CF. |
| BurhanUddin et al. 2011 [20] | Cross-sectional | Villages of four Unions of Kalmakanada Upazila under Netrokona District, Bangladesh. | Families with at least one child under the age of 5, living in different villages of four | 270 families (90 tribal, 180 non-tribal) | Diversity: Cow&rsquo;s milk, Suzi, rice for Cf - and a few used formula milk, goat milk, banana, khichuri, fruit juice.  Frequency:30.4% of Garo and 38% of Non Garo mothers served CF twice, 26.1% of Garo and 37% of Non Garo mothers served 3 times, 43.5% of Garo and 25% of Non Garo mothers served CF 4 times  Advice: 67.6% of Garo and 66.2% Non Garo mothers were advised by doctors. Other sources included mother in law, neighbors, nutritionist, radio/TV  Timing:At 6 months 77.4% had started CF, at 7 months 22% still had not started. Many mothers in both groups started CF before 6 months. |
| Das et al. 2008 [45] | Cross-sectional | Bangladesh | Under-5s from Bangladesh Demographic and Health Survey 2004. | 6005 children | Factors: Barriers - Lack of education/Residence in rural area/Household wealth Index either poor or poorest/Lack of knowledge on CFP; Promoters - Higher number of antenatal visits/Education levels/Household wealth index either rich or richest |
| Faruque et al. 2008 [2] | Mixed methods | Bangladesh | Nationwide. 26,424 0-5 year olds from 151,372 household members. | 26,424 children | Timing: The number of infants starting CF hadincreased substantially with 76% in the current set of data, although CF was often early.  Factors: Promoters - Nutritional interventions e.g. BINP |
| Goudet et al. 2011 [25] | Cross-sectional | Four Dhaka slums, Bangladesh | Pregnant mothers and healthcare workers. | 104 participants | Diversity: Suji and khichuri were mentioned as CF foods.  Preparation: Some women without gas connections were unable to cook meals for their children  Factors: Barriers - Inappropriate care due to breast milk inadequacy, flooding, poverty |
| Goudet et al. 2011 [24] | Cross-sectional | Ten *bosti* slums in Dhaka, Bangladesh. | Mothers aged 19-41, with at least one child aged <3 years and previously exposed to floods. | 78 participants | Diversity: CF included suji, kichuri; also sweets, ice creams, fruits such as apples, oranges and grapes although this was not observed - only reported  Timing: Often inappropriate and introduced too early (before 6 months)  Factors: Barriers - Working mothers/Lack of knowledge by alternate caregivers/Illness of caregiver/Time allocation/Residing in area that floods/lack of food security/inability to work during floods/inadequate breast milk production; Promoters - Increased assets and savings |
| Hackett et al. 2015 [26] | Cross-sectional | Two rural areas in North West Bangladesh and two north-western, rural districts (Rajshahi and Pabna). | Adolescent girls and young women between the ages of 15 and 23, who are SoFEA club members. | 70 mothers | Diversity: CF foods included - Fruit juice, cow&rsquo;s milk, formula milk, water, horlicks (malt). Fruit, vegetables. Khichuri (lentils, rice). Rice. Biscuits. Meat and Fish.  Advice: Religion, doctors, family  Preparation: 67% emphasised safe preparation and storage of child&rsquo;s food  Hygiene: Only four participants emphasised hand washing as important  Timing:63% believed solid foods should be started at 6 months, but reported that &lsquo;softer&rsquo; foods may be given before 6 months  Factors: Barriers - Poor knowledge of IYCF recommendation/Poor interpretation of IYCF recommendations/Insufficient breast milk/Birth complications limit early initiation of breastfeeding/Maternal illness after birth/spoiled breast milk "due to bad air"/Islamic rules giving age cut-offs for breastfeeding/Family members may feed juices when parent is ill; Promoters - sons are fed higher quality CF |
| Hajeebhoy et al. 2013 [44] | Cross-sectional | Dhaka and Chittagong, Bangladesh | Former government leaders; representatives from NGOs, businesses, and religious and academic institutions. | Four focus groups and 13 in-depth individual interviews with 47 thought leaders | Advice: The media  Factors: Barriers - When doctors lacked knowledge they were a barrier to improved IYCF. Food availability and workplace constraints (facility of paid maternity leave) also barriers. |
| Hanif et al. 2013 [19] | Mixed methods | Bangladesh. | Samples from Bangladesh Demographic and Health Surveys based on multi-stage stratified sampling; all households with children under 24 months. | Number of Households: 1993-1994; 9,681. 1996-1997; 9099. 1999-2000; 10,268. 2004; 10,811. 2007; 10,819. 2011; 18,000. | Diversity: Minimum dietary diversity was 25.2% (95% CI: 23.4, 27.0) in 2011 BDHS. Regarding iron, 47.6%(BDHS 07) and 52.8% (BDHS 11) consumed iron-rich food during the past day.  Frequency: MMF was 81.0% (95% CI: 79.2, 82.9) and 64.5% (95% CI: 62.6, 66.4) in BDHS 07 and BDHS 11  Timing: Proportion of infants aged 6–9 months that were breastfed and received CF was 69.2% (95% CI: 64.7, 73.7), 74.2% (95% CI: 70.1, 78.3), and 67.1% (95% CI: 63.2, 71.0) in BDHS 04, BDHS 07, and BDHS 11 |
| Haque et al. 2016 [27] | Cross-sectional | One slum (Mirpur), Dhaka, Bangladesh | Mothers having child aged 6 to 12 months from slum area of Mirpur, Dhaka, Bangladesh | 125 mothers | Diversity: Foods used for CF - Cow&rsquo;s milk/Goat milk, Rice/Rice Powder/Suzy, Infant formula cereals, Fruits, Khichuri, Pulse (dal), Meat, Fish, Egg, Vegetables. Rice/Rice powder/Suzy most popular at 64.8%, Khichuri second most popular at 60.8%.  Frequency: At 6 to 8 months, 44.4% had CF 3-4 times/day, 30.6% 5-6 times/day and few infants only 1-2 times/day/more than 6 times/day. At 9-11 months, 52.0% had CF 5-6 times/day compared to 46.9% at 11-12 months  Preparation: Most mothers prepared infant&rsquo;s food separately. 13.6% were giving family food as per WHO recommendations.  Timing: 47% introduced CF at 6 months, 28.8% before 6 months, 22.4% introduced at 7-8 months, 1.6% after 8 months. |
| Helen Keller International 2009 [18] | Cross-sectional | Bangladesh, | Mothers of 7,000 infants aged 6 - 11 months, and on nearly 64,000 children aged less than 5 years old. | Mothers of 7,000 infants aged 6 - 11 months, and on nearly 64,000 children aged less than 5 years old. | Diversity: Gruel, Fish, egg, dal, green leafy vegetables, and yellow or orange fruit or vegetables were used for CF  Timing: Figure 3 demonstrates timing of starting certain foods. Gruel was sometimes given before 3 months, family foods generally begin at 3-6 months. At 6 months 20% of infants were given family food as a main dietary component, rising to 56% at 9 months and reaching 87% at 12 months.  Factors: Barriers - Poor maternal education/Poor paternal education/Father's occupation (agricultural occupations had improved nutrition)/Residence in rural area/Household wealth index- poor/poorest; Promoters - Father's occupation (agricultural occupations had improved nutrition)/Higher household wealth/Residence in urban area/Improved maternal education |
| Kabir et al. 2012 [6] | Cross-sectional | Bangladesh | Ever-married mothers aged 15-49 of at least one child aged 6-23 months | 1728 children | Diversity: All WHO food groups mentioned (Fish, meat and egg were combined. Overall 41.9% of 6 - 23 months met MDD; this figure was 19.8% for 6-11 months.  Frequency: 81.06% of 6-23 month-olds met minimum meal frequency. 66.17% of 6-11 months olds met MFF , 77.78% of 12-17 month olds, 93.55% of 18 - 23 month olds met MF.  Timing: 71% of children 6-8 months had been introduced to CF  Factors: Barriers - Poor maternal education/Poor paternal education/Father's occupation (agricultural occupations had improved nutrition)/Residence in rural area/Household wealth index- poor/poorest; Promoters - Father's occupation (agricultural occupations had improved nutrition)/Higher household wealth/Residence in urban area/Improved maternal education |
| Kamruzzaman et al. 2009 [28] | Cross-sectional | Three villages of Muradnagar Upazila under the district of Comilla, Bangladesh. | Purposeful sampling of fifty-four [50] families, having children less than two years, | 54 families with children less than two years of age. | Diversity: Rice, shuji, khichuri, cow's milk, formula, biscuit, lentil soup, fruit, vegetables, fish, egg, meat, "usual family food", honey were used for CF.  Timing: 30 out of 54 children (56%) started weaning within 6 months, 13 (24%) 6-12 months, 1 between 11-18 months. 10 out of 54 were not weaned at time of interview. |
| Karim et al. 2012 [29] | Cross-sectional | Villages of Dhamrai, Dhaka, Bangladesh | Mothers in the selected villages | 320 mothers | Diversity: Khichuri/ Hotchpotch was taken by 49.7%), Suji by 21.5%, mixed food by 30.7%; also rice, vegetable, fish, meat  Timing: Out of 320, 239 (91.6%) weaned their babies at within 6 months of life |
| Khan et al. 2011 [30] | Cross-sectional | Pediatrics Mymensingh Medical College Hospital, Bangladesh. | Mothers with children under 2 years old.Four hundred (400) consecutive children were enrolled from eight upazillas of Mymensingh district who were selected randomly | 400 children | Diversity: 18% had Khichuri, 24% cow's milk with suji, 37% Luta, barley, suji; 3% cerelac, 2% banana, 3.7% mixed such as rice with dal, fish, meat or vegetables  Timing: Complementary feeding was timely at the end of 6 months in 35.8%, early weaning <6 months in 44.5%, delayed >7 months in 15% |
| Khatoon et al. 2011 [17] | Cross-sectional | OPD, Dhaka Medical College Hospital, Bangladesh. | 259 children aged 6-23 months of either sex attending the paediatric outpatient department (OPD)Another mothers. | 259 mother-child pairs | Diversity:Dairy, carbohydrate, animal protein, vegetable protein, oil/fat, vit-A rich veg/fruits, other veg/fruits were used as categories and for CF  Frequency: 2.4 was the mean number of times semi-solid or solid-foods given in previous 24 hours  Advice: Over half heard about CF from hospital, clinic, healthcare facility, or healthcare provider  Timing: CF was occurring in all age groups investigated - 6-8 months, 9-11 and 12-23; unclear when it was initiated |
| Khatun et al. 2012 [46] | Cross-sectional | Sobhanbag (government colony), Panthopath (Green road residential area) and one slum area (Rayer bazar) Dhaka, Bangladesh | Mothers of infants aged 2-12 months. | 104 mothers of eligible IFYC | Timing: The study discusses whether mothers had knowledge of the 'optimum weaning time' but does not mention when this is  Factors: Barriers - Low socioeconomic status, if household wealth index status is poor or poorest. Lack of knowledge on CFP timing; Promoters - Socioeconomic status, if household wealth index is rich or richest |
| Kimmons et al. 2005 [31] | Cross-sectional | 9 villages in a rural area (Matlab, Comilla), Bangladesh | 6-12 month olds and their mothers | 135 infants | Diversity: Foods used for CF - rice, wheat, legumes, vegetables (numerous local varieties of tubers, melons, and greens), fruits (guava, banana, mango, coconut), milk, eggs, and fish  Frequency: The mean total number of meals per day was 2.6 ± 1.9  Timing: Children at 6-8 months and 9-12 months were having CF feeds, but this was not broken down into percentages  Factors: Maternal education positively linked to energy intake from CFs |
| Mahejabin et al. 2013 [32] | Cross-sectional | Four selected semi urban villages at Dhamrai Upazilla health complex Dhaka, Bangladesh. | The sample was selected from Srirampur, Dautia, Kalampur and Sutipara of Dhamrai upazilla, the subjects were the breast fed mothers having infants up to 1 year of age. | 320 mothers of infants up to 1 year of age. | Diversity: Khichuri/Hotchpotch was fed to 49.7%, Suji to 21.5%, and mixed food to 30.7%. Cow's milk was also given.  Frequency: At6-9 months: those who were not breast fed were given liquid/soft foods 4-6/day. At 9-12 months those who were not breast fed were fed 6-8 times in a day.  Timing: 62% were fed with CF at 6-9 months and 60.5% were fed with CF at 9-12 months. No infants given CF before 6 months.  Factors: Barriers - Workload/Illness of mother/Lack of Knowledge; Promoters - Knowledge on appropriate CFP |
| Mihrshahi et al. 2010 [41] | Cross-sectional | Bangladesh | Country-wide, mothers ever married (aged 10-49) of 0-23 month old children. | Weighted sample of 2482 children | Timing: 62.3% of infants 6 to 9 months of age were receiving complementary food; 42.5% of those under 6 months were exclusively breast fed, which the authors suggest mean many were given foods or other drinks  Factors: Barriers - Residence in rural area/Lack of education; Promoters - Mothers who had between 3-6 antenatal visits had marginally significant higher rate for timely CFP, urban residence |
| Moore et al. 2006 [33] | Cross-sectional | Five villages in Gazipur, a rural area of Bangladesh. | Five villages where a parenting program for mothers of children under 3 years of age was to be implemented. | Convenience sample of 54 caregiver-child (8-24 months) pairings | Diversity: 61% were fed kitchuri (which sometimes included meat), 53% fruit, 43% rice and 28% eggs; vegetables were not mentioned.  Timing: Mean age to be give solids: 6.8 months, with commencing at 5 - 6 months and some at 12 - 14 months  Factors: Barriers - Specific caregiver behaviours can contribute to poor mother-child interaction in CF; some children demonstrated a lack of interest in eating; Promoters - Responsive behaviours and practice |
| Islam et al. 2008[34] | Cohort | Peri-urban community of Dhaka, Bangladesh. | healthy, breastfed children 8-11 months of age | 18 healthy children (8 boys and 10 girls) | Diversity: Semisolid diets including rice powder  Frequency: Mean CF was 3.2 feeds per day. All had CF >2 times per day at home. |
| Nguyen et al. 2013 [16] | Cross-sectional | Ethiopia, Vietnam, Bangladesh | Baseline survey (&lsquo;Alive and Thrive&rsquo;) included 4400 households in 20 selected subdistricts of mothers with 6-24 month child | 1211 mother-child pairs | Diversity:Uses WHO categories; Grains, roots and tubers, flesh foods, eggs, dairy products, legumes and nuts, Vitamin-A rich fruit and vegetables and other fruit and vegetables. 31.1% of children achieved MDD.  Factors: Barriers - Higher number of children in the family; Promoters - Maternal dietary diversity strongly correlated with child dietary diversity/Maternal education |
| Owais et al. 2016 [15] | Cohort | Two rural sub-districts of Kishoreganj, Bangladesh | Pregnant women in their 7th month of gestation, with follow-up of their offspring scheduled to occur at 3, 9, 16 and 24 months of age. | 2400 mother-child pairs | Diversity: Foods were categorised into the WHO groups. 16% achieved MDD  Frequency: 74 % of infants in the study received at least four meals over a 24 h period  Timing: CF began ≤4 months for 7 %, at 5–6 months for 49 % and at ≥7 months for 44 %  Factors: Barriers - Worse infant diet quality was associated with worse household food security status; Promoters - Infants living in more food-secure households were more likely to receive a minimally acceptable diet at 9 months |
| Paul et al. 2014 [35] | Cross-sectional | Pediatric department (indoor and outdoor) of Sir Salimullah Medical College Mitford Hospital, Dhaka, Bangladesh. | Specific Attendees of hospital Department ;6-24 months old children and children under six months (if was on complimentary feeding) included. | 400 mother/child pairs | Diversity: Kichuri, Suji, Fruit, Meat, Eggs, Pulses, Beans, Rice, Milk, Vegetables were all used for CF.  Frequency:Frequency was appropriate amongst sick children in 206 (56.4%); inappropriate in 159 (43.6%). Frequency amongst non-sick children was appropriate in seven (50%); inappropriate in seven (50%).  Advice: Advice came from Relatives (25%), Qualified doctors (15.3%), Neighbors (14.5%), also Mother in law, husband, television, other family members, other health workers, friends, newspapers, others  Preparation: A blender machine was used for preparing complementary foods by 29 (7.55%)  Timing: 48.4% of mothers initiated CFP at an appropriate time; 51.6% did not  Factors: Barriers - Lack of knowledge/Education/Discouragement from family members/Cultural Factors/Percieved inadequacy of breast milk/Child's behavioural response/Religion/First Born Children; Promoters - Education of nutrition by health workers/ Adequate production of breast milk |
| Rahman et al. 2016 [36] | Cross-sectional | Two rural sites composed of villages from the Basail and Delduar sub districts, within Tangail, Bangladesh | Mothers of children aged 6-24 months and the infant selected from 2 rural sites, | 45 semi structured observations [12], video observations [12], in-depth interviews [17], and focus group [3] | Diversity: Foods used for CF included Kichuri, Suji (semolina), Shemai and Eggs, Fish, Boiled Rice, Cow's Milk, Vegetable Curry, Leafy Vegetables  Preparation: It was done by the mother. 7/12 did not prepare special foods for their children.  Hygiene: Mothers were aware they needed to cover food and food spoils quicker in the summer; 6 knew signs of spoiled food; 10 knew how washing foods and utensils provided safety  Factors: Barriers - Having more than one child/Work pressure/Collecting and using fuel for reheating/Lack of knowledge; Promoters - Convenience, knowledge of CF |
| Rasheed et al. 2011 [14] | Mixed methods | One rural area (South Harina Village of Lohagara Sub- district) in Chittagong and three slum areas (Badda, Saat Tola, and Mirpur) in Dhaka, Bangladesh. | Mothers, fathers, grandparents and other stakeholders included in FGD and informant meetings, of 6-23.9 month old children from areas of high stunting in urban or rural environments. | 484 individuals | Diversity: Foods used for CF - Fruits, leafy green vegetables, pumpkins, eggs, fish, khichuri. Meat, liver and fish were sometimes eaten. MDD - 23.6% consumed food from at least 4 food groups  Frequency: 59% of 6-11 month olds and 59.8% of 12-24 month olds received the recommended frequency for breastfed children  Advice: Sources were village doctors, trained doctors, grandmothers and other family members, traditional birth attendants, neighbours  Preparation: Foods were prepared other family member's foods; few mothers washed off spices, or chopping/grinding foods to make them appropriate  Timing:At 6-8 months, 90.4% were fed solids/semisolids. CF was introduced when babies were days old &ldquo;when the milk did not come in&rdquo; or at 2 to 3 months &ldquo;when the baby cried due to hunger.&rdquo;  Factors: Barriers - Men procure the food, therefore little control over what is available/Lack of knowledge and understanding/Animal-source foods were perceived as inappropriate/Child's response to food given/Time restraints due to chores/Perception breastmilk is inadequate; Promoters - Child behaviour, knowledge of CF, use of local officials |
| Rawat et al. 2014 [13] | Cross-sectional | Twenty sub-districts (*upazillas*) in rural Bangladesh that are part of BRAC's Essential Health Care programme. | Cluster randomized sampling of mothers of 1600 children age 6-11 months in rural Bangladesh. | 1600 mothers-infant pairs | Diversity: Foods used for CF - Meat, Fish, Vitamin A rich foods (yam, pumpkin, orange etc), Green leafy vegetables, Rice, pulses, lentils, eggs and iron rich foods; fortified formulas were given. MDD - <14% met MDD or ate iron rich foods.  Frequency: Meal frequency was of CF was consistent with recomendations; 6% met criteria for minimum acceptable diet. |
| Saha et al. 2008 [37] | Cohort | Matlab; a rural district, Bangladesh. | Sample from the Maternal and Infant Nutrition Intervention in Matlab (MINIMat) study included infants who were born between May 2002 and December 2003 and had completed their 12-month follow-up in December 2004 | 1343 infants | Diversity: Water, fruit juice, other sugary liquids, cow milk, solid & semisolid foods (rice with lentils) were used for CF  Timing: 4 infants were given solid foods <2 months; 1.4% at 3 months, 4% at 4 months, 13.2% at 5 months; at 6 months, 43.9% had solid foods; at 10 months, 94.1%; at 12 months, 92%  Factors: Barriers - Poor household food security; Promoters - Better prior feeding practices/Infant's male sex/Mother's higher education/Higher wealth index/Good household food security. |
| Saleh et al. 2014 [40] | Cross-sectional | Four slum areas (Tejgoan, Rayerbazar, Beribadh and Jafrabad) Dhaka, Bangladesh | Mother-child pairs living in location | 120 pairs | Frequency: Frequency of meals was divided between Exclusively Breast Fed (EBF) children and non-EBF children - overall rates were not available but are recalculated - at 6-8 months 14/23 (61%) were fed 2 times a day, 9/23 3 times a day (39%). At 9-11 months, 17/24 (71%) were fed 2 times a day, 7/24 (29%) 3 times a day. At 12-23 months, 28/73 (38%) were fed 2 times a day, 45/73 (62%) 3 times a day.  Hygiene: Inadequate handwashing, cleaning of utensils, handwashing of children, covering of food, and reheating of food was identified  Timing: For non EBF children, CF started before 6 months in 78/93 children (84%) and at 6 months in 15/93 chidren (16%)  Factors: Barriers - Lack of knowledge on CFP, poor psychosocial care |
| Salim et al. 2012 [38] | Cross-sectional | Paediatrics department of Sir Salimullah Medical College and Mitford Hospita, Dhaka, Bangladesh. | Purposive sampling method total of 455 mothers having their children of less than two years of age Visiting the hospital Department. | 455 mothers | Diversity: Foods used for CF -cooked suji, barley, rice powder mixed with cows milk or powdered milk, khichuri, rice, fish, meat, eggs, vegetables, Cerelac, artificial baby foods  Timing: 24% started CF at 6 months. For 47.8% CF was early at <6 months, for 28.4% delayed from >7 months.  Factors: Barriers - Lack of education; Promoters - Maternal secondary education |
| Sarkar et al. 2003 [42] | Cross-sectional | Seven slum areas in Dhaka, Bangladesh. | Households with children younger than 24 months living with foster parents or biological parents. | 46 foster child/parent pair 82 pairs of biological mother/child aged <24 months. | Timing: 58% foster children recieved gruel as introductory feed in first 4 months of life, compared to 14% of non-foster controls  Factors: Barriers - Lack of knowledge of CF practices, Poverty, Being a foster child |
| Sultana et al. 2014 [39] | Cross-sectional | Kapasia under Gazipur district ,Bangladesh | Mothers of 150 children under the age 2 purposively selected living in Kapasia. | Mothers of 150 children aged under 2. | Diversity: CF foods included rice, khichuri, meat, fish, egg, potato, fish, green leafy vegetables, cow/goat milk, suzi, dal, pumpkin, ripe papaya, palong shak, kochu shak, pui shak, banana, carrot.  Frequency: Information on frequency was given by different foods, but not as a composite for number of meals per day.  Hygiene: 76% of mothers had appropriate handwashing, 11.3% washed both hands and utensils appropriately; 91.5% used soap after using the toilet.  Timing: 16.9% introduced CF before 6 months; in 83.1 it was timely. |
| Zongrone et al. 2012 [8] | Cross-sectional | Bangladesh | Country-wide household population with at least one child aged 0-23 months | 2096 mother-child pairs | Diversity: All 7 WHO categories were used for CF. 51.46% consumed Fe rich foods, however food names are not stated. MDD was 45.16%.  Frequency: For children of 6 - 23 months, the proportion that achieved MMF was around 85%.  Timing: Around 80% introduced CF at 6-8 months |
